# Barriers to and Facilitators of Implementing Suicide Risk Screening in Pediatric Primary Care: A Qualitative Study

**DOI:** 10.1016/j.acap.2026.103252

**Published:** 2026-02-14

**Authors:** Erin R. Orlins, Katherine Winner, Shilpa Sangvai, Amanda J. Thompson, Alice A. Gaughan, Delaney Marsalek, Xiaodan Hu, Sooyoung Kim, Jeffrey A. Bridge, Cynthia A. Fontanella, Ann Scheck McAlearney

**Affiliations:** Center for Suicide Prevention and Research (ER Orlins, AJ Thompson, D Marsalek, JA Bridge, and CA Fontanella), Nationwide Children’s Hospital, Columbus, Ohio; Department of Mental Health (K Winner), Dayton Children’s Hospital, Dayton, Ohio; Boonshoft School of Medicine (K Winner and S Sangvai), Wright State University, Dayton, Ohio; Department of Pediatrics (S Sangvai), Dayton Children’s Hospital, Dayton, Ohio; The Center for the Advancement of Team Science, Analytics, and Systems Thinking (AA Gaughan, X Hu, S Kim, and AS McAlearney), The Ohio State University College of Medicine, Columbus, Ohio; Department of Psychiatry and Behavioral Health (JA Bridge and CA Fontanella), The Ohio State University College of Medicine, Columbus, Ohio; Department of Pediatrics (JA Bridge and AS McAlearney), The Ohio State University College of Medicine, Columbus, Ohio; Department of Family and Community Medicine (AS McAlearney), The Ohio State University College of Medicine, Columbus, Ohio

**Keywords:** mental health, pediatric primary care, suicide screening, youth

## Abstract

**Objective:**

Universal suicide risk screening in pediatric primary care settings is lacking, despite the endorsement of this practice by several national organizations. This study explores barriers to and facilitators of implementing universal suicide risk screening in pediatric primary care clinics, with a focus on clinics that had not yet adopted this practice.

**Methods:**

We conducted a qualitative study consisting of semistructured interviews with primary care clinicians and support staff involved in suicide risk screening, risk assessment, and disposition planning. Participants were recruited from 15 pediatric clinics in the Midwestern United States. Interviews were audio-recorded, transcribed verbatim, and analyzed using thematic analysis.

**Results:**

From 55 interviews, we identified barriers at 3 levels: clinic, provider, and patient. Clinic-level barriers included time, workflow, and staffing. Provider-level barriers included lack of training/self-efficacy, frustration at not being able to offer solutions, and burnout. Patient-level barriers included irritation with repetitiveness of screening questions, concerns about honesty and openness, parent/caregiver buy-in, and discomfort and stigma in discussing the topic of suicide. Facilitators included perceived value and effectiveness of screening, comfort with screening, clinic buy-in, a team approach to care, providing privacy, and providing context. Suggestions to enhance these suicide prevention practices were also described.

**Conclusions:**

Overall, clinicians and support staff reported seeing value in implementing suicide risk screening in pediatric primary care. Our findings underscore the importance of reducing barriers and enhancing facilitators associated with implementing suicide risk screening, risk assessment, and disposition planning to expand suicide prevention efforts to more youths.

Suicide is a leading cause of death among US youth, with rates among those aged 12 to 17 increasing 54% between 2004 and 2023.^1^ In 2023, 1555 youths in this age group died by suicide.^1^ A national survey found that 81% of pediatricians had a patient attempt or die by suicide.^2^ Notably, 70% of youth who died by suicide had seen a primary care provider in the year prior; only 30% saw a mental health professional.^3–5^ Recognizing the critical role of primary care in suicide prevention, in March of 2022, the Joint Commission and the American Academy of Pediatrics issued new recommendations for universal suicide risk screening—screening all patients regardless of presenting complaint or behavioral health concerns—for youth beginning at age 12.^6^ To support implementation, the American Academy of Pediatrics and American Foundation for Suicide Prevention, in partnership with the National Institute of Mental Health, released the *Blueprint for Youth Suicide Prevention*.^7^

The importance of universal suicide risk screening for youth is well-established, as “targeted screening” or screening only individuals at high risk is insufficient.^8^ Youth rarely discuss mental health or suicide unless directly asked,^9^ and depression screening alone (eg, via the Patient Health Questionnaire-9 [PHQ-9]) misses nearly one third of youth at risk for suicide.^10^ Many youth who screen positive for suicide risk have no known mental health diagnoses.^11^ Although most pediatric providers support suicide risk screening,^12,13^ more than 85% do not implement universal screening with a standardized suicide-screening tool,^2,14^ citing barriers such as limited time, inadequate training, and lack of follow-up resources.^13^

Validated suicide–specific screening tools, such as the Ask Suicide-Screening Questions (ASQ), are brief to administer,^12^ and research shows no iatrogenic effects from suicide risk screening.^15^ However, implementation in pediatric primary care remains challenging. These settings are often busy, and effective screening requires coordination among physicians, nurses, and support staff. Positive suicide risk screens also require follow-up procedures, including risk assessments to determine suicide severity, and disposition planning to identify appropriate and safe next steps for care.

Despite growing attention to suicide risk screening in pediatric primary care, few studies have examined the factors that influence implementation in real-world settings. We aimed to identify barriers and facilitators to adopting universal screening in pediatric primary care clinics. The study extends prior work^13^ by including a larger interdisciplinary sample across multiple clinics serving diverse populations and geographic regions, none of which had implemented universal suicide risk screening. A secondary purpose was to use these insights to inform the implementation of a universal suicide–-screening clinical pathway. Understanding barriers and facilitators is critical to improving the uptake of evidence-based practices to identify at-risk youth and enhance outcomes.

## Methods

### Study Design

We conducted a qualitative study using semistructured interviews to explore perceived barriers and facilitators to implementing universal suicide risk screening in pediatric primary care.

### Participants and Recruitment

Participants were recruited from 15 pediatric primary care clinics in a Midwestern state participating in the Stepped Approach to Reducing Risk of Suicide in Primary Care (STARRS-PC) study, a hybrid I stepped wedge effectiveness-implementation trial. The parent study evaluates a population–based quality improvement intervention (ie, STARRS-PC) compared to treatment as usual to reduce suicidal behavior among youth in pediatric primary care. STARRS-PC includes a clinical pathway for youth at elevated suicide risk consisting of universal suicide risk screening, assessment, and triage using the Brief Suicide Safety Assessment (BSSA),^16^ and, when indicated, follow-up transitional care. This qualitative study was conducted during the treatment-as-usual phase, when suicide risk screening practices varied across participating clinics; 10 of 15 clinics reported screening, most commonly using the PHQ rather than a suicide-specific tool. Criterion–based purposeful sampling was used to recruit participants. Clinic leadership first identified all staff whose roles involved suicide risk screening, risk assessment, and disposition planning. Recruitment was monitored to ensure representation across key roles, with 4 to 5 participants from each clinic, including physicians, nurse practitioners, nurses, clinical managers or coordinators, behavior and mental health specialists, administrators, and office staff. The study was approved by the Institutional Review Board at Nationwide Children’s Hospital and followed the Consolidated Criteria for Reporting Qualitative Research guidelines.^17^

### Data Collection

A semi–structured interview guide assessed current suicide risk screening and management practices, as well as perceived barriers and facilitators to implementing universal screening. Questions were informed by a comprehensive literature review and input from a multidisciplinary research team, including pediatricians and qualitative methods experts. Given the exploratory nature of the study, no formal theoretical framework or hypothesis–guided question development; instead, questions broadly addressed barriers, facilitators, and clinic processes relevant to suicide risk screening. The guide was piloted with 5 clinicians who conduct suicide risk screenings but were not affiliated with study clinics and refined based on feedback. Nine master’s and doctoral-level researchers (including E.R.O., A.J.T., and A.S.M.) conducted the interviews, which lasted approximately 1 hour, and were held either in-person or via telephone/video call between October 2023 and May 2024. Participants provided informed consent before the interview, received $50 remuneration, and interviews were audio-recorded and transcribed for analysis.

### Data Analysis

We explored clinicians’ and support staff’s perspectives using inductive thematic analysis.^18^ After interviews were completed, the transcripts were analyzed by 3 coders who immersed themselves in a subset of transcripts and developed an initial codebook focused on barriers and facilitators to suicide risk screening, assessment, and disposition planning. Following discussion and refinement, the revised codebook was applied to all transcripts, with regular meetings to discuss emergent codes. Atlas.ti 25 was used to support the coding and analysis. After coding was complete, the team met with the broader study group to identify patterns and define overarching themes. To enhance trustworthiness, we conducted member checking with 2 participants to assess whether the themes reflected their experiences, acknowledging that themes may not resonate with all participants.

## Results

We interviewed 55 participants from pediatric primary care clinics across urban and rural settings (Table 1). Participants’ comments were used to identify key themes and subthemes related to barriers, facilitators, and implementation suggestions for universal screening.

### Barriers to Suicide Risk Screening, Risk Assessment, and Disposition Planning

Participants identified barriers across 3 levels: clinic, provider, and patient (Table 2).

#### Clinic-Level Barriers

Three main clinic-level barriers were described by participants: time, workflow, and staffing. Many participants described the lack of time due to already busy schedules and short appointment slots. One noted, *“Time is probably the biggest thing… if they’re doing a regular visit plus addressing any suicidality in the same visit, that’s an additional 20 minutes. Especially if they flagged really high [on a screener]—we have to call [the] psychiatric department.”* (28). Others shared concerns about workflow disruption. One participant explained, *“It’ll definitely slow things down because it’s one more tool and its questions that are sensitive questions. And anything that is answered in a concerning or semi-concerning way needs follow-up.”* (43). Staffing limitations also emerged as a barrier. As one participant described, *“There was concern about identifying suicidality because we didn’t have the staff to complete the safety plan… if we ask about suicide and they say yes, and we don’t have someone to write a safety plan, then what do we do?”* (41).

#### Provider-Level Barriers

Provider-level barriers included lack of training, frustration with limited treatment options, and burnout. Many participants felt unprepared to meet the needs of youth who were identified at risk for suicide through screening. One noted, *“We sometimes feel like psychiatrists with 10-minute slots and no psych residency.”* (15). Others expressed frustration when unable to offer timely solutions. As one put it, *“When we ask these questions—okay great. Now what am I going to do? Put you on a six-month waitlist for a counselor or a ten-month waitlist for psychiatry? That’s not a good answer.”* (26).

Third, some participants described provider burnout as a challenge. As one reflected, *“…when we have provider burnout, the provider is not able to go through all that needs to be done… so, you can imagine with a parent that doesn’t want to be part of screening and even have to go convince them. Well, a physician that’s burnt out, you know what? Just leave it alone. Let’s move to the next patient.”* (33).

#### Patient-Level Barriers

Participants described several patient-level barriers to suicide risk screening, assessment, and disposition planning practices, including irritation with repeated screening questions, concerns about honesty, lack of parent/caregiver buy-in, and stigma around discussing suicide. Youth frustration with repeated questions was commonly reported. One participant noted, *“They say… I’ll say it nicely— ‘I already effing answered these questions.’ They get irritated. They get irritated.”* (51). Concerns about honesty were also raised, particularly when parents were present. As one participant explained, *“I’m sure there are some [kids] out there that just say no to everything…they’re afraid to say yes…they don’t wanna tell the parent that they’re suffering from anxiety or depression.”* (16).

Lack of parent or caregiver buy-in was another common barrier. As one interviewee summarized, *“Sometimes parents don’t necessarily feel their child needs help.”* (9) Others highlighted logistical challenges, such as competing demands and limited time. One participant explained, *“Some [parents] might not be as motivated because they might have several other children and multiple jobs…they don’t have a lot of time to take their child to mental health resources that are referred to them.”* (11).

Stigma and discomfort discussing suicide were frequently cited as barriers. One participant noted, *“There’s such a stigma and fear associated with talking about it [mental health].”* (35) Others mentioned parental discomfort, especially with younger adolescents. As one explained, *“There’s also going to be some parents that don’t like the word suicide being given to their child—-especially when we start a lot of these screenings at 12. They don’t want to think their kid can have depression. They don’t want to think their kid can have suicidal thoughts. And they would prefer their kid don’t know what suicide is.”* (29).

### Facilitators of Suicide Risk Screening, Risk Assessment, and Disposition Planning

Based on interviewees’ comments, we identified 6 facilitators: perceived value and effectiveness of screening; comfort with screening; clinic buy-in; team-based approach; providing privacy; and providing context (Table 3).

#### Perceived Value and Effectiveness of Screening

Clinicians and support staff widely recognized the importance of universal suicide risk screening, often linking it to their core motivation for working in pediatrics. As one explained, *“We’re all in the pediatric world because we love it… suicide is close to our hearts. We try everything we can to help the families.”* (1). Many noted the urgency of screening given the prevalence of suicide in their communities: *“It’s a definite need that we’ve all witnessed in our community—the kids struggle.”* (30). Others emphasized that youth often do not disclose mental health concerns unless directly asked: *“It’s not really spoken about unless asked… it’s kind of masked.”* (31). Screening was also viewed as a strategy to reduce suicidal behavior through early identification and follow-up: *“We can identify these kids early, especially if we start it at age twelve…then see them back more frequently so somebody’s following them…ultimately something I think is extremely valuable in the long run of essentially decreasing attempts.”* (28).

#### Comfort With Screening

Participants noted that comfort with screening—among patients, parents, and staff—was a key facilitator. One shared, *“It’s just part of routine care… no different from asking them if they’re having any pain or if they’re having any other problems.”* (11). Others observed that youth are increasingly open to discussing mental health: *“Patients are more accepting of being asked these questions.”* (38). and *“The younger generation—they are much more ready to discuss mental health.”* (47). Parents also expressed relief when the topic was addressed: *“They’re like, ‘I’m glad you brought this up. I’m glad somebody’s asking. I’m glad this is being discussed.’”* (20).

#### Clinic Buy-In

The importance of having buy-in from everyone in the practice was noted as another facilitator. One participant reflected, *“So once the physicians are bought in, it will happen. Because we are going to expect it to happen and then the staff… make sure that they’re on board, and that’s worked.”* (27).

#### Team Approach

Clinicians and support staff also noted that a team approach to suicide prevention was an important facilitator. As one participant explained, *“As long as everybody’s on the same page, I think it’ll make it easier for everybody. Everybody does it the same way, everybody goes down the line…So, I think a team approach is the best way to do it.”* (9).

#### Providing Privacy

Participants highlighted how providing youths with privacy was important when administering the screener and discussing suicide risk. One participant summarized, *“On the provider side, just trying to get them away from their parent to talk to them one on one and let them know they’re in a safe space, you can tell us.”* (25).

#### Providing Context

Finally, participants noted that parental psychoeducation before introducing suicide risk screening for their child could provide context and potentially increase acceptance of the process. As one participant commented, *“Explanation—just having an understanding of what that process is, I think, makes parents more agreeable to follow-up.”* (30).

### Suggestions for Suicide Risk Screening, Risk Assessment, and Disposition Planning

Participants offered 7 suggestions for implementing suicide prevention practices: standardizing processes; ensuring training; establishing expectations; providing education materials; offering electronic screening; integrating screening data with electronic health records; and co-locating behavioral health services (Table 4).

#### Standardize Processes

Clinicians and support staff noted that having clear protocols and consistent practices would be important in implementing universal suicide risk screening and follow-up processes. One participant stated, *“I think any time you can create a flight guide… in medicine, we’ve taken a book out of pilots’ playbooks and really tried to create checklists and tried to create redundancy in how we do things. And when you have redundancy and you have a checklist that happens with every patient, every time, the likelihood of missing patients is drastically decreased.”* (18).

#### Ensure Training

Participants also described the importance of providing training opportunities to build confidence and competence. One participant reflected, *“I’m still not 100% comfortable and I feel I could definitely use more tools and education on being able to tease out what’s immediate and what something that, maybe it’s not an active plan, but they’re thinking about it.”* (2).

#### Establish Expectations

Clinicians and support staff highlighted the importance of framing universal suicide screening as a practice to reduce resistance and stigma, thereby normalizing its use in the clinic. One participant described, *“…as we have these conversations on a more regular basis, it normalizes these conversations to some extent. And it’ll spur conversations between patients and parents.”* (18).

#### Provide Education Materials

Providing families with accessible, visible information about mental health and suicide risk screening was also recommended. As one participant noted, *“I think that if we were able to just have resources out in the lobby that parents and teens were able to visually see while they were waiting, that may give resources in the local area, or just more education on the importance of being screened for depression, anxiety, for suicide.”* (38).

#### Offer Electronic Screening

Many participants endorsed electronic screening as a helpful tool, citing benefits such as increased privacy, honesty, and reduced language barriers. One noted, *“The computer is nice because it keeps them steps away from a family member…they have some privacy if they need it.”* (8). Another observed, *“Children are more apt to be truthful if they give them the choice to go up to the screen.”* (1). A third added, *“I really wish that the iPad also had the screening on it…we can immediately click a button and change the language if there’s a language barrier.”* (10).

#### Integrate Electronic Screening and Electronic Health Record (EHR) Data

Participants noted that integrating suicide risk screening with the EHR could reduce errors and enhance clinic workflows. One explained, *“If it’s completed on paper and someone else is entering that data into the system, it’s easier to make an error…”* (53). Another highlighted the value of data access: *“Oh, being able to see the timeline of it. Having quick, easy access to that timeline…I think would be very helpful rather than having to scan through physical paper.”* (40).

#### Co-Locate With Behavioral Health

Participants suggested several ways in which co-locating behavioral health services within primary care could support suicide prevention practices, including providing the ability to conduct warm handoffs, streamline workflows, and potentially increase patient trust, leading to a greater willingness to seek care. One explained, *“It gets rid of this question of who do I go to? How do I respond to this? … So, I think that from a provider’s perspective, having access to behavioral health professional, counselor, or social worker within integrated within the primary care department reduces a lot of the barriers and a lot of the hesitancy and a lot of the questions about well, how do I address this?”* (53).

## Discussion

Through in-depth interviews with pediatric primary care clinicians and staff, we identified key barriers and facilitators to implementing suicide risk screening, assessment, and disposition planning. Universal suicide risk screening was generally viewed as acceptable, consistent with prior research in primary care^12,13^ and other medical settings, including pediatric specialty clinics,^19^ emergency departments,^20^ and inpatient units.^21^

Notable barriers to implementation included clinic-level challenges such as time, workflow, and staffing, which are commonly cited in the literature.^22,23^ Although suicide risk screening takes about 30 seconds and follow-up assessment takes 10 to 15 minutes,^24^ time burden remains a concern. However, most nurses and pediatricians implementing universal screening reported minimal workflow disruption^12^ and identified opportunities to streamline implementation despite competing demands.

Consistent with prior research, we identified provider-level barriers, including limited mental health training. Training is critical for the successful implementation of universal suicide risk screening as primary care providers with adequate knowledge of suicide risk assessment are 5 times more likely to screen adolescents.^14^ Pediatric residency programs increasingly recognize the importance of suicide risk assessment and care management training,^25^ suggesting that incorporating this content into residency curricula may be an effective starting point. Mental health training may also benefit providers as those reporting suboptimal care for youth with behavioral/mental health conditions are nearly twice as likely to experience moral distress.^26^ Another key barrier was difficulty connecting at-risk youth to appropriate resources, reflecting the national shortage of child psychiatrists.^27^ Strategies to address this include establishing agreements with external mental health providers, including telehealth, co-locating behavioral health services within primary care, developing pediatric psychiatric consultation teams, and providing ongoing training to support providers in managing youth while awaiting specialty care.

Overcoming patient-level barriers is also essential and will require careful attention. Youth irritation with repeated screening and stigma around discussing suicide may lessen as routine suicide risk screening becomes normalized at medical visits and mental health discussions become more common. Providing youths and caregivers with a brief rationale for screening and education about suicide may further reduce irritability and stigma. Consistent with prior research on family perceptions of suicide risk screening in emergency departments, concerns about honesty—particularly youths’ reluctance to disclose mental health issues to parents—highlight the need to balance caregiver involvement with respect for adolescent privacy and autonomy.^28^ Further research is needed to identify strategies that optimally address both adolescent privacy and caregiver involvement.

Regarding implementation strategies, electronic screening may enhance privacy and has been shown to be both feasible and acceptable.^29^ Suicide risk screening toolkits provide scripts clinicians and staff can use to introduce the screening to patients and families,^30^ helping to establish context. Standardization was another key recommendation. A structured suicide risk screening pathway for outpatient primary and specialty care has been developed^12^ and may serve as a model. This 3-tiered process includes brief suicide risk screening using tools, such as the ASQ and BSSA, followed by a disposition plan for those who screen positive. The BSSA categorizes youth into 3 levels—low risk, further evaluation needed, and imminent risk—guiding subsequent care. Providers have reported high satisfaction, feasibility, and comfort with the ASQ and BSSA,^31^ supporting the potential value of this pathway in pediatric primary care. Future work should utilize implementation mapping to link the barriers and facilitators identified in this study to targeted implementation strategies.

## Limitations

This study has several limitations. First, although participants’ roles involved suicide risk screening, assessment, and disposition planning, their perspectives may not reflect those of all clinic staff engaged in these processes. Second, clinics were in a single state, which may limit generalizability. Third, clinic leadership had already agreed to participate in the parent suicide intervention study, and staff may therefore have been especially motivated. Fourth, we captured only clinicians’ and support staff’s perspectives; future research should include youths and parents. Finally, while participants noted system-level factors such as insurance constraints and mental health workforce shortages, this analysis focused on clinic-, provider-, and patient-level barriers and facilitators, and broader structural issues warrant further study.

## Conclusion

Given the potential public health impact of universal suicide risk screening, pediatric primary care clinics must address anticipated barriers and strengthen facilitators to support successful implementation and improve patient care and outcomes.

## Figures and Tables

**Table 1.** Characteristics of Interviewees and Clinics

	n	%
Interviewee role (n = 55)		
Physician	15	27.3
Nurse practitioner	10	18.2
Registered nurse or related nursing role	12	21.8
Clinical manager or coordinator	6	10.9
Behavioral and mental health specialist	4	7.3
Administrator or office support staff	8	14.5
Participating clinic information (n = 15)		
Location		
Urban	7	46.7
Rural	8	53.3
Size		
Small	5	33.3
Large*	10	66.7
Type		
Hospital-affiliated practice	5	33.3
Large multispecialty network	4	26.7
Community health center	4	26.7
Independent practice	2	13.3
Co-located behavioral health		
Yes	8	53.3
No	7	46.7
Percentage of patients aged 12–17 currently screened for suicide risk		
None (0%)	5	33.3
Some (1–33%)	3	20.0
About half (34–66%)	1	6.7
Most (67–99%)	4	26.7
All (100%)	2	13.3
Among those that currently screen (n = 10), frequency with which patients are screened for suicide risk		
Every visit	5	50.0
Annually (well visits)	2	20.0
Other (eg, clinician discretion)	3	30.0
Among those that currently screen (n = 10), version of suicide risk screener used (could be more than 1)		
Any version of the Patient Health Questionnaire	9	60.0
Ask Suicide-Screening Questions	1	6.7
Columbia-Suicide Severity Rating Scale	1	6.7
Ages and Stages Questionnaire	1	6.7
Standardized protocol for risk assessment for patients identified as at risk for suicide		
Yes	3	20.0
No	12	80.0

* Large is defined as > 600 patients seen annually.

**Table 2.** Barriers to Implementing Suicide Risk Screening in Primary Care Settings

Challenge	Verbatim Comments
*Clinic-level barriers*	
Time	*“I think, sometimes, as providers, we don’t have enough time to really ask all the questions that need to ask and really confirm that they’re getting the help that they need… that’s one of the biggest things as far as our problems with suicidality and depression and mental health in our office.”* (3). *“The time, I will say sometimes is difficult because when you have the kids that screen positive that opens up a whole other area that you need to kind of sit down and tease out and kind of figure out what’s going on. And when you only have a 30-minute block and you have all of these things that you’re supposed to be covering… you kind of have to figure out what you need to do to make it work…”* (2).
Workflow	*“It’s going to push out appointments, make it late, decrease patient satisfaction especially, as a whole. It’ll take longer to room them, especially when you have parents who want to talk about multiple issues. Especially when you get into mental health.”* (19). *“…if you have a child that is going through this [mental health concerns], it’s really time consuming. If I mean, of course, it’s worth every minute, but it’s just your other patients can get very backed up. So that’s a real problem.”* (4).
Staffing	*“So, for example, we have walk-in appointments where a 13-year-old could come in for a sore throat [and] screen positive on a suicide screen. We have one nurse with four docs in the office, in the middle of the winter. We have no capacity on the inside.”* (27). *“I mean in a clinic that’s already very busy, staffing is always a problem. I think that’ll have to be addressed for sure.”* (40).
*Provider-level barriers*	
Lack of training/self-efficacy	“… *this isn’t our specialty. It’s not our skill set. Maybe the newer physicians feel differently. But I know - we’ll call them more seasoned physicians because it’s nicer - they say all the time, ‘I wasn’t trained for this. I wasn’t trained for mental health. I* was *trained to help a kid grow.’ Yet, here we are, serving as mental health providers.”* (29). *“I feel comfortable asking, but then sometimes if there is a risk, I don’t feel I got a lot of practice or training in residency about the next steps after that. Like I am familiar with safety plans. I’m familiar with what to do and kind of risk stratification, but I don’t always feel the most comfortable with it.”* (42).
Frustration at not being able to offer solutions	*“Our problem wise, is that we have nowhere for them after we ask them. After they identify, it seems these kids hit a roadblock… we don’t have anything in our area to help these kids immediately.”* (14). *“…we want to help these kids. But we want to have a way to help these kids. It’s terrible to identify a problem and then not be able to offer a solution. That’s what’s frustrating… Not the screening.”* (17).
Burnout	*“…providers are now expected to delve into a lot at visits that used to be much simpler than they are now. So, a Well Check. Now we fill out these screens and if the child fills it out concerning for any mental health issues, then we need to delve into that. And I think provider burnout can lead to people just not feeling they have the energy to delve into it the way we should.”* (15). *“I would say burnout in general is gonna affect the quality of care that everybody is getting, right? If a provider is exhausted and overstressed and overworked and all these other things, I don’t think that they’re going to be doing their jobs to the best of their ability…I think that that could mean things like screening tools don’t get done, right? Just because if they’re overstressed and they’re seeing a ton of patients and they’re in and out. Maybe they don’t feel there’s time for that.”* (35).
*Patient-level barriers*	
Irritation with repetitiveness	*“Honestly, they’re getting tired of it. We asked these questions every visit and they get tired of answering these questions. They get tired of filling out the PHQ-9 (Patient Health Questionnaire-9) forms when they answer ‘yes’ to one of these questions.”* (14). *“You always have some of those patients who don’t want to do it or refuse or get annoyed because they already answered it.”* (5).
Honesty and openness	*“Probably the most challenging is…just kind of getting all the information out of the kid. Because I think once you’re like, ‘OK, well, this is a big deal, and I have to tell your mom…’ Then the wall goes up, right? They’re scared … you can visibly see that they don’t want to tell you as much anymore and you’re like, ‘OK, but I need you to tell me… to be honest so I can help you the best.’”* (10). *“Sometimes, you know, they’ll present, and they’ll look a lot more down or depressed than what they’re putting on paper and you know that maybe they’re just not admitting how they’re really feeling on the paper.”* (21).
Parent/caregiver buy-in	*“When it comes to children, the problem is sometimes the parent. The problem is the children rely on the adults around them so heavily…Where if the parent is not engaged, or feels that it’s not true, or it’s a farce, then it can make it very difficult for the child ….”* (47). *“So, some parents are really in tune to those issues, but some are just blinded because their child could not have those issues. …So, it’s very, I think down the middle in this community, where, you know some are like, ‘Yes, it’s an issue. I’ve seen it, I worry about my child,’ and other parents are just very much like ‘Pshh, not my kid. They have everything they want; they can never be depressed.’”* (8).
Stigma and discomfort in discussing suicide	*“Yeah, mental health is still definitely a stigma. And more so from my experience in rural communities versus urban communities. And there’s still definitely pushback, even discussing depression in the office, there’s sometimes parents who push back on that.”* (18). *“But again, it’s that stigma where when you call to make the appointment, the parents are like ‘Ohh, she’s fine. She’s fine. She’s fine.’ So, I think that’s one of the things that we really need to work on is the stigma with the parents about mental health, that it’s not really…it’s not really, a flaw, it’s a disease, you know?”* (12).

**Table 3.** Facilitators of Suicide Risk Screening Implementation

Facilitator	Verbatim Comments
Perceived value and effectiveness of screening	*“I think it’s [screening is] needed…. We do catch a lot before it becomes that issue, I believe. And I think it also gets kids talking about it, you know. That it is a problem and that it opens us up to a conversation if they’re having struggles.”* (17). *“I mean the relatively young kids who we see suicide problems, I mean 11- and 12-year-olds— it used to just be kind of older kids. And I think that’s the scary part for a lot of parents is you can tell when there’s a major life event. But some of these kids are having suicidal thoughts and suicidal events or attempts over things that I think many of us as adults would perceive as relatively minor things. And so how do you anticipate it? How do you prevent it? How do you-? It’s complex. It’s difficult. It’s scary. All those things.”* (36).
Comfort with screening	*“I don’t see a push back…My perception is, for the most part, I think, kids are definitely open.”* (22). *“I’m pretty comfortable. I’m used to asking patients if they have any plans and intent. And if they don’t have intent, are they thinking about it or are they harming themselves?”* (48).
Clinic buy-in	*“I think it takes provider buy-in. And we have to be the sellers of this. We can’t kind of have attitude of, oh, this is some dumb thing we have. If the providers do that, nothing’s going to work.”* (36). *“So, I think just having everybody on the same page. And they can kind of look out for positive screens. If they have concerns when they’re rooming the patient or when they’re discharging the patient, you know. I think just having open communication is really helpful that way.”* (42).
Team approach	*“When you have team-based care, there’s a lot of eyes. When you have a lot of eyes, it’s a lot better because the odds of missing things or it being dropped [fall]. A lot of eyes help. When you have your front desk direct to the kiosk, the kiosk is done. The front desk is aware, the screen automatically populates that they’re at risk, identifies a number. Then they go back, you have an MA (medical assistant), then possibly a nurse. Then you have the provider, and then they call BH (behavioral health). Everybody’s included. So, it’s nice that way.”* (46). *“I mean team approaches are necessary because if someone’s lacking on their job it takes another member of the team to say, ‘hey, this is what we do’…we can encourage each other and remind each other. And if somebody’s falling short in doing routine screening, then it can be brought up as a concern or issue. But also having a team approach allows for double checks. So, say if registration doesn’t hand out that paper with the screening on it for the child to fill out in the waiting room, it doesn’t matter. We’re a team. And so, the patient doesn’t have the paper. I’m the backup plan in that I can just bring it up online and do it with the patient myself. So, it’s a check and balance situation.”* (11).
Providing privacy	*“As they get older, they ask the parent to step out of the room. So, you’re more likely to get truthful answers then. You know, we don’t want mom to know this, so they don’t talk about it.”* (24). *“They may have been less likely to divulge or be open or honest if a parent was nearby.”* (45).
Providing context	*“I think it’ll be an adjustment. But I think once they [parents] realize that it’s not just them…we’re screening everyone. It’s not because your kids said something and it gave us red flags. We’re screening everyone. I think parents will be fine.”* (3). *“Most of them are fine with them [screens]. I mean you always get that couple patients who are like ‘Oh my gosh, another paperwork.’ But most are fine. And especially when we take the time and explain,” Look, I’m doing this because I care about your kid’s wellbeing and safety.’ …they’re perfectly fine with it.”* (10).

**Table 4.** Suggestions to Improve Suicide Risk Screening and Care Management

Suggestion	Verbatim Comments
Standardize processes	*“I would be excited for it not only to be available but streamlined and made a specific protocol. More consistency across the board would be really nice amongst providers.”* (3). *“I would love to have a more structured approach. …I would love to say if they’re four these are the things you should do. If they’re seven, these are the things you do, right? I think when you’re using too much subjective instead of objective, you’re just opening up holes where kids might slip through the cracks… I need that algorithm to say these are the steps you need to take. These are the ones that have been proven to be best outcomes.”* (7).
Ensure training	*“Making sure everyone is trained on it. If the rooming staff is doing this, which it is, I think something that rooming staff could do—asking questions and documenting it in some type of standardized chart. They already have a lot to do. They go from site to site sometimes, and they float. So, it’s important that if we’re doing it at our site that needs to be done at every single site that we have and getting everybody on board from site to site. I think that is definitely a challenge.”* (48). *“I think that if we train our staff properly, I think going into it knowing exactly what they’re doing, what the process is, makes it go a lot smoother when they just kind of get something thrown at them that makes things go poorly. So, I think, up front, clear understanding of what we’re doing, how the process will go, who’s role is what, what we all need to do, a crystal-clear kind of plan. And organization will make things go a lot better.”* (36).
Establish expectations	*“And I think the best way to reduce that push back from patients is to normalize it and make it a regular thing that you do in the office. And try and make it not a big deal as much as you can. Make it a big deal but also make it not something that is abnormal. … So, if they come in and from 11 until 18, we do the same type of thing, and it becomes something that happens in the office and that’s discussed with parents and with patients, it makes it a conversation that we have multiple times.”* (18). *“Just trying to make sure everybody’s comfortable and knows that it’s a normal thing to discuss these issues…”* (32).
Provide education materials	*”Maybe a resource on suicide in teenagers might be helpful sometimes for parents just so that they have some facts. Because I feel not everybody realizes how frequent it is and how much it happens because they don’t see it a lot sometimes in this area. I feel it would be helpful if we have some of that information - a pamphlet or brochure or something.”* (8). *“We don’t give any handouts to our patients to take home, to help educate them on suicide risk. …but I would think it would probably be something that would be very helpful actually in our own clinic.”* (11).
Offer electronic screening	*“I personally like the electronic because papers get missed or papers get lost. So, I think that it’s a safer way to do it. Because then if the paper gets lost and someone else sees it that shouldn’t be seeing it, then that’s HIPAA (Health Insurance Portability and Accountability Act) violation. So personally, I’m all for the electronic.”* (52). *“So, I think getting it electronic. And I think that way we can reach more people and hopefully have the results up front, rather than trying to chase our tail at the end of the appointment.”* (42).
Integrate screening data with electronic health record (EHR)	*“I will say it would be great to have something electronic…it’s quicker in integration into the EMR (electronic medical record) too and scoring it for us. Because if somebody comes in, screens high, we already know before we walk into the room and can go ahead and start that process before we go in there and start a conversation. …I think it would move things along quicker.”* (28). *“I feel if we were able to either have iPads or a kiosk or something for the teens to be able to independently fill out their screens electronically…it wouldn’t be as time consuming to hand out the papers, to collect the papers, put in the results.”* (38).
Co-locate with behavioral health	*“If we could have even somebody come in part time, occasionally peek-in, see some of the kids. I think it would help. It’s very hard to find places for our children to go to. And when you do find a place, it’s taking forever for them to get in, like months to be seen. So, if we had somebody in the facility occasionally that could see some of those kids until we’re able to get that set up and those first appointments are able to be met, I think that would be life changing for those kids, maybe even keeping them out of our ERs (emergency rooms) for psych evals (evaluations)… ”* (8). *“I personally, one, I think, in those moments when I’m not sure which way to go, I have that next level of expertise, right. Just like if I don’t know if this ear needs tubes, I send them to the next level of expertise, right? We’re the jack of all trades, the expert of none. I want that expert level. I want that personal relationship. And I also think when you’re dealing with a mental health issue and there’s all this talk and then nothing that comes to fruition, you don’t believe in it. And I know teenagers, adolescents in particular, they’re very, very concrete. So, when we say this is the plan, we’re gonna help you, we’re building up this positivity. And then the waitlist is four months. Or Mommy can’t even get through to this clinic. And so having that person where I can put a name to a face and that may not be their be all end all, but it’s at least proof in the pudding, that something is gonna happen for this kiddo. Yeah, even if it was just someone that we could call and that we knew each other by name, you know. And that’s not gonna happen all the time. But not having it just feels like a weak link in our care.”* (7).

## What’s new

Clinicians and staff perceived suicide risk screening in pediatric primary care as valuable in identifying youths at risk for suicide. Findings highlight the need to reduce barriers and enhance facilitators to ensure feasible, effective, and sustainable implementation of these practices.
